# Valorization of Plant-Based Food By-Products Through Green Extraction of Bioactive Compounds for Functional Food

**DOI:** 10.3390/molecules31040646

**Published:** 2026-02-13

**Authors:** Cristina-Anca Danciu, Alina-Georgeta Mag, Cristian Stanciu, Livia Vidu, Mirela Stanciu

**Affiliations:** 1 Faculty of Agricultural Sciences, Food Industry and Environmental Protection, Lucian Blaga University of Sibiu, 7-9 Dr. Ion Ratiu Str., 550012 Sibiu, Romania; 2 Faculty of Social Sciences and Humanities, Lucian Blaga University of Sibiu, 5-7 Victoriei Blvd., 550024 Sibiu, Romania; alina.mag@ulbsibiu.ro; 3 Faculty of Sciences, Lucian Blaga University of Sibiu, 5-7 Dr. Ion Rațiu Street, 550012 Sibiu, Romania; cristiandumitru.stanciu@ulbsibiu.ro; 4 Faculty of Animal Productions Engineering and Management, University of Agronomic Sciences and Veterinary Medicine of Bucharest, 59 Marasti Blvd., District 1, 011464 Bucharest, Romania; livia.vidu@usamv.ro

**Keywords:** plant-based food by-products, valorization, functional ingredients, bioactive compounds, green extraction technologies

## Abstract

The revalorization of food processing by-products represents a critical strategy for enhancing resource efficiency and advancing circularity within the food system. This review examines the potential of three major plant-based agro-industrial by-products—fruit and vegetable residues, brewer’s spent grain, and spent coffee grounds—as sources of high-value functional ingredients. These by-products contain bioactive compounds, including dietary fibers, polyphenols, proteins, peptides, oils, and antioxidants, that can be recovered using emerging green extraction and bioprocessing technologies. Conventional extraction methods are progressively being replaced or hybridized with enzyme-assisted, ultrasound-assisted, microwave-assisted, and deep eutectic solvent techniques to improve yield, reduce solvent consumption, and preserve bioactivity. The recovered compounds have demonstrated promising applications as gelling agents (pectin), natural colorants and antioxidants, protein-enriched flours, prebiotic fibers, and bioactive extracts for functional food and nutraceutical formulations. However, challenges persist in standardizing feedstock composition, scaling continuous extraction processes, ensuring safety and regulatory compliance, and generating robust techno-economic and life-cycle assessments to validate sustainability claims. This review synthesizes biochemical composition data, processing pathways, food applications, and regulatory considerations, and identifies research priorities for developing integrated, scalable biorefinery models that valorize food by-products into market-ready functional ingredients.

## 1. Introduction

The global food industry faces an unprecedented waste crisis. Approximately one-third of all food produced for human consumption is wasted, equivalent to 1.3 billion tons annually [1]. This waste generates significant environmental degradation, greenhouse gas emissions, and economic losses.

Fruit and vegetable processing industries’ residues represent approximately 16% of total food by-products, contributing roughly 6% to global greenhouse gas emissions [2]. Brewer’s spent grain represents 85% of the brewing industry’s solid by-products, with global production exceeding 39 million tons annually [3]. Similarly, spent coffee grounds represent a major waste stream from global coffee production, with millions of tons generated annually [4].

The transition toward circular economy models necessitates innovative valorization strategies that align with green chemistry principles [5]. This transformation depends critically on developing sustainable extraction methodologies that preserve bioactive compounds while minimizing environmental impact.

Global food production systems generate substantial volumes of organic residues, a large proportion of which possess nutritional, biochemical, and techno-functional potential [6,7]. However, much of this biomass is traditionally managed through low-value or environmentally burdensome routes such as landfill disposal, incineration, or use as undifferentiated animal feed [8,9]. Recent estimates suggest that 20–30% of globally processed food biomass becomes by-products or co-products [10]. The re-valorization of these materials aligns with the principles of the circular bioeconomy, in which waste streams are reconceptualized not as disposal liabilities but as reservoirs of recoverable, high-value compounds [11,12].

Among numerous agro-industrial waste streams, fruit and vegetable processing residues (FVR), brewer’s spent grain (BSG), and spent coffee grounds (SCG) represent particularly abundant examples that are compositionally rich in biopolymers (fiber, proteins, pectins, lignocellulosic polysaccharides) [13,14], polyphenolic antioxidants [15], lipids [16], and bioactive secondary metabolites [17,18]. These matrices are therefore promising substrates for the development of functional food ingredients, nutraceuticals, and clean-label additive alternatives [19,20].

However, valorization is constrained by feedstock variability [21], a lack of standardized extraction protocols, regulatory ambiguities, and insufficient techno-economic validation [22,23]. Addressing these challenges is essential to transform food residues from waste streams into reliable, safe, and economically viable sources of functional ingredients, thereby supporting climate goals, resource conservation, and innovation in the food and nutraceutical sectors.

The objective of this review is to critically analyze and synthesize current knowledge on the revalorization of major food processing by-products—specifically fruit and vegetable residues, brewer’s spent grain, and spent coffee grounds—as sustainable sources of high-value functional ingredients. The review aims to (i) select representative agro-industrial residues, (ii) characterize their bioactive fractions as targets for recovery, (iii) evaluate green extraction techniques for sustainable valorization, and (iv) identify technological, regulatory, and sustainability-related challenges that must be addressed to enable scalable, integrated biorefinery models within a circular food system.

This review focuses on three abundant and compositionally rich plant-based agro-industrial by-products: fruit and vegetable processing residues (FVR), brewer’s spent grain (BSG), and spent coffee grounds (SCG). These materials were selected due to their global availability, their high content of bioactive compounds such as dietary fibers, proteins, polyphenols, and lipids, and their demonstrated potential for conversion into functional food ingredients. By concentrating on FVR, BSG, and SCG, we provide an integrated perspective linking biochemical composition, green extraction technologies, and functional food applications. Moreover, the work emphasizes the transition from laboratory-scale valorization to industrial implementation, highlighting the need for continuous extraction systems, harmonized safety assessments, and robust techno-economic and life-cycle analyses.

## 2. Literature Review

This literature review analysis synthesizes current research on plant-based food by-products valorization through green extraction approaches to produce functional ingredients. The field represents a rapidly growing intersection of sustainability, food technology, and circular economy principles, with significant applications in functional food development and environmental mitigation.

**Figure 1:**
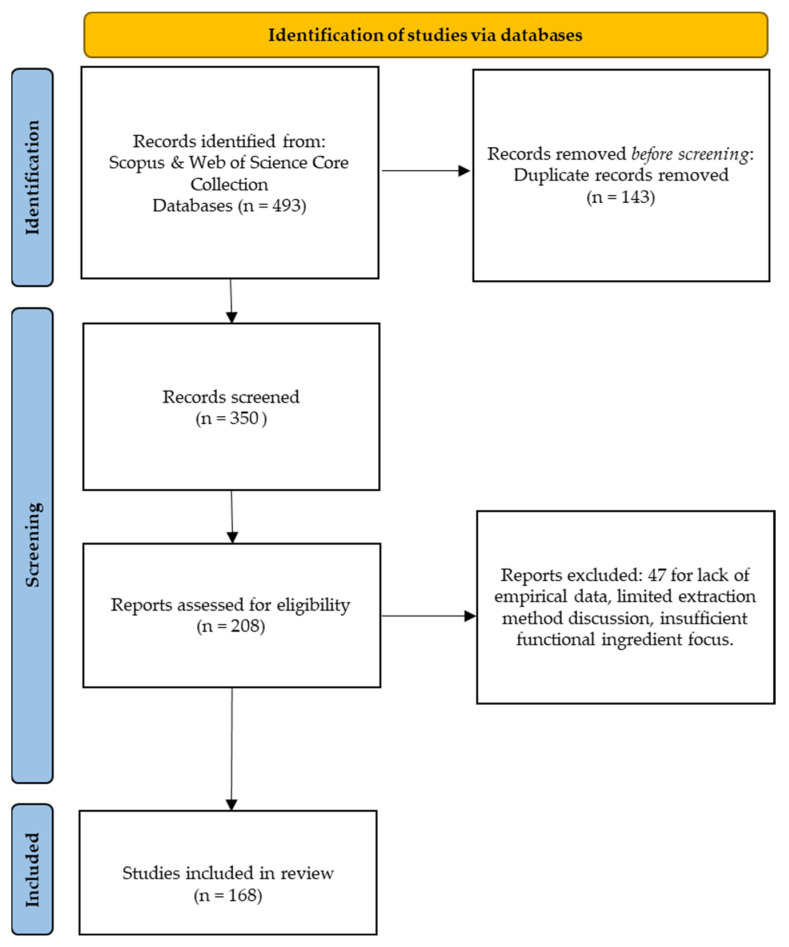
PRISMA flowchart for systematic review, adapted from [24].

The bibliometric analysis was conducted from the second half of September until the first week of December 2025, and followed the Preferred Reporting Items for Systematic Reviews and Meta-Analyses (PRISMA) 2020 guidelines [24] to ensure transparency and methodological rigor (Figure 1). The analysis conducted searches across academic databases, Scopus and Web of Science, using predefined search strategies combining keywords related to food by-products, valorization, green extraction, functional ingredients, and sustainable approaches (“food by-product*” OR “agro-industrial residue*” OR pomace OR peel OR “fruit residue*” OR “spent coffee” OR “brewer* spent grain”) AND “green extraction” OR “sustainable extraction” OR “ultrasound-assisted” OR “microwave-assisted” OR “enzyme-assisted” OR “supercritical” OR “pressurized liquid” OR “deep eutectic solvent*” OR NADES OR “subcritical water” AND “functional ingredient*” OR polyphenol* OR pectin OR fiber OR “dietary fiber”).

To ensure comprehensive coverage of recent scientific developments, the time span was limited to 2020–2025. Studies were included if they: (1) addressed plant-based food by-product utilization; (2) discussed extraction methodologies, particularly green or sustainable approaches; (3) examined bioactive compound recovery; (4) investigated applications in functional food development; and (5) were published in peer-reviewed English-language journals. Studies lacking empirical data, not addressing extraction or bioactive compound applications, or focusing exclusively on non-food sectors were excluded. After duplicate removal, the dataset comprises 161 original research articles, and review papers, indicating that the field is strongly driven by experimental development with a rapidly growing review literature. This distribution reflects the technological maturation of green extraction methodologies and their increasing relevance to applied food, environmental, and materials sciences.

## 3. Composition and Bioactive Potential of Major Plant-Based Food Processing By-Products

Global agro-industrial processing generates significant volumes of plant-based residues, a large proportion of which possess nutritional, biochemical, and techno-functional potential. This review focuses on fruit and vegetable processing residues (FVR), brewer’s spent grain (BSG), and spent coffee grounds (SCG). FVR encompass the peels, pomace, seeds, and pulp generated during fruit and vegetable processing, and thus are highlighted in Table 1. BSG, the major solid by-product of the brewing industry, exhibits relatively consistent composition and is rich in proteins, fibers, and phenolic compounds. SCG, generated in large quantities from coffee production and brewing, contain lipids, phenolics, and polysaccharides with significant antioxidant and prebiotic potential. Focusing on these three residues allows for a systematic comparison of biochemical composition, functional ingredient potential, and green extraction strategies.

### 3.1. Fruit and Vegetable Residues (FVR)

Fruit and vegetable processing residues—including peels, pomace, seeds, and pulp—represent rich sources of bioactive compounds [45]. These materials contain dietary fibers, flavonoids, phenolic acids, antioxidants, polysaccharides, vitamins, and natural pigments. Among these, soluble fibers—particularly pectin—are of high industrial relevance due to their gelling, emulsifying, and water-binding capacities [46].

For citrus by-products specifically, major compounds include D-limonene, carotenoids, and pectin, with phenolic acids such as chlorogenic acid, caffeic acid, and coumaric acids being particularly abundant [47]. Fruit pomace is the most extensively studied and valorized fraction. Notable examples include: pomegranate pomace, containing 13.6% protein and 34.6% neutral detergent fiber; pineapple pomace, with approximately 45% fiber; and citrus pomace, which typically contains 20–30% fiber [38].

Apple by-products contain significant levels of phlorizin, chlorogenic acid, and epicatechin, demonstrating both antioxidant and cardioprotective properties [48].

Tomato processing residues contain lycopene, β-carotene, and lutein—compounds exhibiting potent biological activities relevant to cancer prevention and oxidative stress reduction [49].

### 3.2. Brewer’s Spent Grain (BSG)

BSG comprises approximately 60% carbohydrates (50% dietary fiber), 30% proteins, and 10% lipids, along with significant mineral content and bioactive polyphenols [50]. Compared with FVR, BSG exhibits greater compositional consistency due to standardized brewing operations, making it an attractive feedstock for controlled biorefinery processes [51].

The protein fraction consists mainly of water-insoluble hordeins and glutelins with molecular weights ranging from <5 kDa to >250 kDa [42].

Key phenolic compounds in BSG include ferulic acid, p-coumaric acid, and caffeic acid, largely bound to the lignocellulosic matrix [3]. These compounds exhibit documented antioxidant, anti-inflammatory, and hepatoprotective properties. Additionally, BSG contains substantial levels of arabinoxylan and β-glucan—prebiotic polysaccharides with documented health-promoting effects [21].

### 3.3. Spent Coffee Grounds (SCG)

SCG represent concentrated sources of bioactive compounds, including chlorogenic acid, caffeine, and diverse phenolic compounds [40]. SCG typically contain 6–16% protein, 10–18% lipids, and 60–70% total dietary fiber (mainly cellulose, hemicellulose, and lignin) on a dry basis [42,43,44,50]. The reported higher fiber values in the literature generally refer to isolated insoluble fractions rather than whole SCG and should be interpreted accordingly [43]. Major fatty acids recovered include palmitic (C16:0) and linoleic (C18:2) acids. SCG polysaccharides consist primarily of galactomannan and arabinogalactan chains, with glucuronic acid remaining attached even after roasting and brewing processes [51].

Coffee by-products contain 6–16% protein, 10–18% dry basis lipids (notably diterpenes such as kahweol and cafestol, which exhibit antioxidant and anti-inflammatory properties), melanoidins formed during roasting, and up to 94% dietary fiber (cellulose, hemicellulose, lignin), positioning them as nutrient-dense waste streams [52]. The phenolic fraction contains chlorogenic acids, though their stability is influenced by roasting severity and brewing conditions. The chlorogenic acid content—reaching concentrations of 5–36 mg/g dry weight—makes SCG particularly valuable for functional food applications [53].

## 4. Valorization of Plant-Based Food By-Products into Food-Grade Functional Ingredients

The valorization of FVR, BSG, and SCG offers a scientifically robust and industrially feasible pathway toward sustainable food systems, climate-neutral processing, and resilient biobased value chains, strongly supporting global circular economy and sustainability strategies.

The valorization of fruit and vegetable residues (FVR), brewer’s spent grain (BSG), and spent coffee grounds (SCG) into food-grade ingredients has demonstrated strong technological maturity and growing industrial relevance. Recovered compounds such as pectin, proteins, polyphenols, and dietary fibers exhibit well-established functional properties including gelling, thickening, emulsifying, antioxidant, and nutritional enhancement capabilities (Table 2).

These functionalities enable their successful incorporation into a wide range of food matrices, including bakery products, beverages, dairy and plant-based alternatives, meat analogs, snacks, and functional foods.

### 4.1. Valorization of Fruit and Vegetable Residues: Pectin, Polyphenols, and Dietary Fibers

Fruit and vegetable residues, including peels, pomace, seeds, stems, and trimmings, represent 25–60% of the original raw material depending on the commodity and processing method [77]. These residues are particularly rich in pectin, polyphenols, and dietary fibers, making them high-value feedstocks for food, nutraceutical, pharmaceutical, cosmetic, and packaging industries [78,79,80]. Non-digestible carbohydrates from food by-products exhibited promise for gut microbiota modulation, with immunomodulatory effects supporting disease prevention and health promotion [81]. Polysaccharide incorporation from fruit and vegetable waste demonstrated the ability to modify gut microbiota while enhancing immune function through lymphocyte stimulation and inflammatory response control [82,83]. Citrus by-products demonstrated promising anti-diabetic potential through enzyme inhibition mechanisms [84]. Onion skin waste extracts produced via subcritical water extraction demonstrated significant α-glucosidase inhibition (IC50 = 75.6 ± 43.5 μg/mL), surpassing acarbose (IC50 = 129.5 ± 1.0 μg/mL) in both pure enzyme and cell culture tests without cytotoxicity to human cell lines [85]. Their valorization aligns strongly with circular bioeconomy and zero-waste food system strategies [86].

The preservation of pectin structure and polyphenol bioactivity from fruit and vegetable residues is closely linked to the application of mild, green extraction technologies detailed in Section 5.

#### 4.1.1. Pectin Extraction Technologies and Structural Characteristics

Conventional pectin extraction relies on acid hydrolysis under elevated temperature (80–95 °C) and pH 1.5–3.0 [87]. While effective, this process is energy-intensive and may induce depolymerization, reducing gel strength [87]. Non-thermal or hybrid techniques—enzyme-assisted extraction, ultrasound-assisted extraction (UAE), and microwave-assisted extraction (MAE)—enhance mass transfer, preserve molecular integrity, and reduce acid load [88]. Deep eutectic solvents (DES) have recently emerged as tunable, biodegradable solvent systems capable of the selective solvation of pectic domains while minimizing the co-extraction of unwanted matrix components [89].

#### 4.1.2. Polyphenol Recovery and Antioxidant Applications

Bioactive compounds recovered from fruit, vegetable, and cereal by-products exhibit well-documented antioxidant properties [45]. Polyphenols recovered from grape pomace, citrus peel, berry skins, and tropical fruit residues demonstrate strong radical-scavenging activity, metal-chelating capacity, and inhibition of lipid oxidation [90]. These extracts are increasingly examined as clean-label alternatives to synthetic antioxidants (e.g., BHT, BHA) in emulsified and lipid-rich food matrices [91]. Encapsulation via spray-drying, micro fluidization, or protein–polysaccharide complexation enhances stability and bioavailability [92,93].

#### 4.1.3. Functional Fiber Concentrates and Techno-Functional Properties

High-fiber fruit residues—apple pomace, citrus fiber, carrot pomace—exhibit hydration, swelling, and oil-binding properties that improve texture and water retention in bakery and meat analog systems [94]. Finely milled fiber fractions can enhance viscosity, stabilize emulsions, and increase nutritional value while partially reducing fat content [95].

### 4.2. Valorization of Brewer’s Spent Grain

Functional bread production utilizing BSG represents a promising application within circular economy frameworks [96]. The enrichment of wheat bread with spray-dried and fermented BSG at two addition levels revealed that fermented BSG (FBSG) improved bread characteristics significantly, including increased specific volume, reduced crumb hardness, and restricted microbial growth rates [97]. At 5–10% BSG incorporation, overall sensory acceptability remained comparable to control breads while achieving “high fiber” and “source of fiber” claims according to EU regulations [96]. Functional cookies supplemented with BSG powder (10–40%) demonstrated proportional increases in total polyphenol and flavonoid contents, reaching 4.26-fold and 4.05-fold higher values than controls, with enhanced antioxidant activities measurable by ABTS and reducing power assays [98].

Meat industry applications of BSG reveal promising opportunities for nutritional enhancement [99]. In burger formulations, 15–20% BSG incorporation improved fiber and protein levels while decreasing fat and calories without negatively affecting sensory acceptance [100]. Hybrid meat formulations maintaining protein content while incorporating BSG showed similar consumer acceptability to traditional formulations [101]. However, concentrations exceeding 20% BSG negatively impacted sensory and technological properties, introducing undesirable flavors and altering texture [102]. This identifies the optimal incorporation ranges, which are critical for commercial viability.

BSG phenolic extracts exhibited protective effects against oxidant-induced DNA damage, possibly through iron chelation mechanisms [103]. Melanoidins derived from BSG showed increased antioxidant activity correlating with browning degree development during Maillard reaction, with antioxidant properties increasing as polyphenols bind to melanoidin structures [104]. BSG incorporation into functional foods created products supporting blood glucose management through dietary fiber content and polyphenolic bioactivity [50].

Selective protein and fiber recovery from brewer’s spent grain relies heavily on enzyme-assisted, ultrasound-enhanced, and fermentation-integrated extraction approaches, detailed in Section 5.

#### 4.2.1. Protein Recovery and Functional Performance

BSG proteins (primarily hordein, glutelin, and albumin fractions) present functional attributes including emulsification, foaming, and gelling [101]. Brewer’s spent grain proteins demonstrate considerable functional potential as plant-based protein sources [51]. Alkaline extraction followed by isoelectric precipitation has been widely applied; however, pH extremes may induce structural unfolding and loss of solubility [105]. Novel extraction systems—protease-assisted hydrolysis, ultrasound-enhanced solubilization, and DES-based protein fractionation—achieve higher yields with preserved functionality [106]. A comparison of extraction methods revealed that pulsed electric field (PEF) extraction achieved ~90% improvement in protein recovery compared to conventional alkaline extraction [107].

The extracted BSG protein isolate (EverPro) demonstrated 100% solubility compared to 22% and 52% for pea and soy isolates respectively, with superior foaming capacity and minimal sedimentation activity [108]. Enzymatic modification through proteolytic hydrolysis produced functional protein hydrolysates exhibiting antioxidant, anti-inflammatory, and angiotensin-I-converting enzyme inhibitory activities [109].

#### 4.2.2. Dietary Fiber and Arabinoxylan Recovery

Brewers spent grain contains substantial arabinoxylan and β-glucan content with significant prebiotic potential [110]. The extraction of arabinoxylan from BSG through simultaneous saccharification and fermentation achieved approximately 21% arabinoxylan solubilization from unprocessed BSG, with concentrated samples containing 99% soluble arabinoxylan demonstrating bifidogenic effects and enhanced short-chain fatty acid production in in vitro fecal fermentation trials [110]. Sequential extraction and attenuated total reflection–Fourier transform infrared spectroscopy monitoring enabled integral recovery of major BSG components, with reactive extraction approaches enabling the simultaneous extraction and tunable functionalization of hemicellulose [111]. The highest arabinoxylan-containing sample increased Lactobacillus levels approximately twofold and produced bifidogenic effects with 3.5-fold increases in Bifidobacterium levels, alongside enhanced acetate (*p* = 0.018) and propionate (*p* < 0.001) production [112].

#### 4.2.3. Fermentation and Integrated Bioprocessing Routes

Solid-state fermentation of BSG using *Bacillus subtilis* and other bioprocessing strains enhanced nutritional profiles dramatically [113]. Fermentation with WX-17 strain increased total metabolite content substantially, with 2-fold increases in total amino acids and 1.7-fold increases in unsaturated fatty acids, alongside remarkable 5.8-fold increases in antioxidant quantity [114,115]. Fermented BSG incorporation into pasta resulted in superior protein digestibility, quality indices, and essential amino acid profiles compared to native BSG [116]. In vitro digestion studies demonstrated enhanced phenolic antioxidant activity persistence and protective effects toward oxidative stress in Caco-2 cell cultures [113].

#### 4.2.4. Food Formulation Applications of Brewer’s Spent Grain

BSG-derived flours have been incorporated into bread, extruded snacks, pasta, plant-based meat analogs, and beverages [117]. The principal formulation challenge is mitigating the graininess and bitterness associated with fibrous and phenolic fractions. Strategies include particle-size reduction, wet fractionation, and enzyme-assisted softening [118].

### 4.3. Valorization of Spent Coffee Grounds: Lipids, Antioxidants, and Dietary Fiber

Emerging evidence supports application of SCG-derived extracts as antioxidant stabilizers in emulsions, natural colorants, and prebiotic dietary fibers [119].

Spent coffee grounds and cocoa shell valorization through combined ohmic-accelerated steam distillation and supercritical CO_2_ extraction demonstrated significantly enhanced efficiency compared to traditional methods [102]. Recovery rates for BSG reached 89% for antioxidants, 91% for phenolic acids, and 90% for polyphenolic compounds, with notably high yields of p-coumaric acid (95%), gallic acid (94%), and ferulic acid (82%) [120].

Coffee by-products demonstrate particular promise in skincare applications due to their chlorogenic acid and caffeine content supporting photoprotective and anti-aging properties [121]. Their implementation as natural ingredients in cosmeceuticals has shown safety, stability, and skin improvement potential, positioning coffee waste as a sustainable alternative to synthetic ingredients [121].

#### 4.3.1. Lipid Extraction Technologies and Applications

Oil extraction from SCG has traditionally used Soxhlet or hexane-based systems; however, concerns around solvent residues and environmental impact have driven interest in supercritical CO_2_ extraction and enzyme-assisted lipid release [122]. SCG oil has been evaluated as a natural antioxidant additive, emulsion stabilizer, and nutritional lipid ingredient [123]. However, diterpenes can raise LDL-cholesterol when consumed unfiltered, necessitating their fractionation or selective removal for food-grade applications [124,125].

#### 4.3.2. Polyphenols, Melanoidins, and Antioxidant Functionality

Polyphenol-rich extracts from SCG demonstrate radical-scavenging, ferric-reducing, and lipid oxidation inhibitory activity, supporting their use as clean-label antioxidants in emulsified foods, plant-based spreads, and bakery products [126]. SCG extracts demonstrated DPPH-scavenging capacities of 3089–3136 μmol TE/100 g oil and FRAP values of 4324–4383 μmol TE/100 g oil, levels comparable to or exceeding conventional antioxidant extracts [127].

Melanoidins provide Maillard-derived antioxidant coloration, but their high molecular weight complicates their characterization and bioavailability predictions [128,129]. Research trends increasingly favor encapsulation and protein–polyphenol complexation to enhance stability [129].

#### 4.3.3. Dietary Fiber from Spent Coffee Grounds and Prebiotic Potential

SCG contains both insoluble and soluble fibers that display water-holding, oil-binding, and viscosity-building properties. Recent in vitro digestion studies suggest the selective fermentation of SCG polysaccharides by Bifidobacterium and Lactobacillus, indicating possible prebiotic effects [112]. However, human clinical validation remains limited, representing a significant research opportunity [130].

Given the lipid-rich and antioxidant-dense nature of SCG, advanced solvent-based and supercritical extraction technologies play a decisive role in their valorization, as outlined in Section 5.

### 4.4. Comparative Perspective and Outlook

The valorization of FVS, BSG, and SCG offers complementary and synergistic pathways toward sustainable food ingredient production.

From a functional perspective, FVR are particularly suited for applications requiring soluble fibers and natural antioxidants, such as bakery products, dairy alternatives, and functional beverages. However, their high moisture content and compositional variability—driven by cultivar, seasonality, and processing conditions—necessitate stabilization and standardization steps prior to industrial use. Regulatory acceptance of pectin and fiber-rich fractions is generally favorable, although concentrated phytochemical extracts may require additional safety assessment depending on purity and intended use [131].

Compared with FVR, BSG exhibits greater compositional consistency but presents challenges related to its lignocellulosic recalcitrance and potential negative sensory impacts at high inclusion levels [132]. While many BSG-derived ingredients fall within existing regulatory frameworks, novel protein or peptide fractions may require additional authorization depending on their processing intensity and final application.

Compared with FVR and BSG, SCG offer unique opportunities for lipid recovery and antioxidant extraction; however, their valorization is constrained by potential safety and sensory concerns, including residual caffeine, process-induced contaminants, and bitterness [133]. Regulatory acceptance of SCG-derived ingredients remains more limited, often requiring careful fractionation and toxicological evaluation to ensure food-grade compliance.

The effective recovery and functional performance of these valorized compounds are strongly influenced by the extraction and bioprocessing strategies employed, which are critically discussed in the following section.

## 5. Green Extraction and Bioprocessing Technologies for the Valorization of Plant-Based Food By-Products

**Figure 2:**
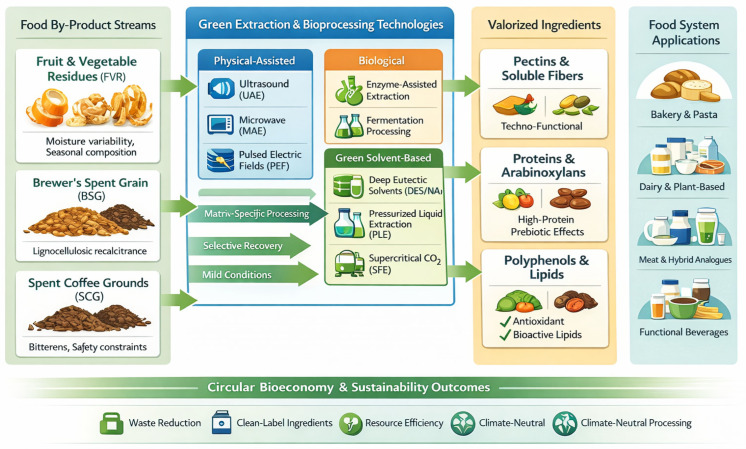
Green technologies for the valorization of FVR, BSG and SCG.

The selection of appropriate green extraction and bioprocessing technologies is critical for preserving functionality, ensuring food-grade safety, and enabling the industrial scalability of valorized ingredients. Building on the valorization strategies for FVR, BSG, and SCG (outlined in the previous section), the transition toward sustainable food systems necessitates the replacement of conventional organic solvent-based extraction methods with environmentally benign alternatives (Figure 2).

A recent bibliometric analysis identified that, between 2015 and 2025, ultrasound-assisted extraction (UAE) and deep eutectic solvents (DES)-based processes emerged as the most frequently applied green extraction techniques [5]. The advantages and limitations of green extraction technologies are depicted in Table 3.

### 5.1. Ultrasound-Assisted Extraction (UAE)

Ultrasound-assisted extraction utilizes acoustic cavitation to enhance mass transfer and cell disruption, enabling the efficient recovery of bioactive compounds from matrix-bound states [130,131], increases solvent penetration, and accelerates mass transfer [132]. At the laboratory scale, UAE achieves high recovery of pectins, polyphenols, and proteins from fruit and vegetable residues (FVR) and brewer’s spent grain (BSG) at relatively low temperatures, preserving molecular integrity. For instance, citrus pomace extraction via UAE improved composite performance scores by 207% while reducing energy consumption by 77% [139].

UAE has shown enhanced yields of pectin, phenolics, and proteins at lower temperatures, preserving molecular integrity and reducing hydrolytic degradation. The application of UAE to citrus peel extraction achieved total phenolic contents of 40–260 mg GAE/100 g dry matter, with hesperidin comprising up to 58 mg/g peel [133]. For brewer’s spent grain, ultrasound-assisted extraction enhanced extraction efficiency when combined with deep eutectic solvents, demonstrating phenolic yields of 0.516 mg GAE/L at 30 °C [134].

Industrial Considerations: Scaling UAE presents challenges, including uneven cavitation distribution in large volumes, potential localized overheating, and the need for robust reactor design [140]. High-intensity ultrasound can also induce the partial depolymerization of polysaccharides or degradation of delicate polyphenols if process parameters are not carefully controlled. Continuous-flow systems and optimized sonication profiles are therefore recommended for industrial implementation.

### 5.2. Microwave-Assisted Extraction (MAE)

Microwave-assisted extraction applies radiofrequency energy to the rapid heat extraction of solvents and substrates, accelerating solvent penetration and compound recovery [5]. MAE heats polar biomolecules and water molecules directly, reducing extraction time and solvent consumption. Laboratory-scale studies demonstrate rapid extraction, high yield, and reduced solvent usage. For example, microwave extraction of orange peel with 80% ethanol at 373 K for 6 min yielded 7.2 ± 0.1 mg GAE/g total phenolics, outperforming conventional methods [141].

Industrial Considerations: Scaling MAE introduces risks of localized overheating and thermal degradation, particularly for heat-sensitive compounds like high-molecular-weight pectins, flavonoids, and proteins. Maintaining homogeneous energy distribution, controlling power input, and employing continuous-flow or hybrid reactor systems are critical to preserve functional integrity at large scale [134]. MAE proves particularly effective for thermally robust phenolics and polysaccharide extraction from spent coffee grounds, enabling the recovery of polysaccharides with lower molecular weight while preserving glucuronic acid attachment to arabinogalactan chains [135], with careful adaptation required for lab-to-industrial translation.

### 5.3. Enzyme-Assisted Extraction (EAE)

Enzymes (e.g., pectinases, cellulases, proteases) selectively hydrolyze matrix components, releasing bound bioactives under mild conditions. Enzymatic hydrolysis enables the targeted recovery of protein hydrolysates and bioactive peptides from plant by-products [136]. Laboratory studies show high selectivity, enabling the recovery of protein hydrolysates, peptides, and soluble fibers from BSG and FVR with preserved bioactivity. EAE combined with ultrasound can produce protein isolates with superior emulsifying and foaming properties compared to chemical extraction [50].

Industrial Considerations: Enzyme cost, stability, and process control are significant constraints. Maintaining optimal pH, temperature, and reaction time is essential to prevent enzyme denaturation or over-hydrolysis. Scale-up often requires immobilized enzymes or continuous bioreactor designs to maintain yield and functional quality while controlling costs [136].

### 5.4. Deep Eutectic Solvents (DES)

DES systems (e.g., choline chloride–urea) provide the tunable solvation of polyphenols, proteins, and pectins while maintaining low toxicity. The primary limitation is viscosity, which complicates downstream purification [140].

Natural deep eutectic solvents represent a revolutionary green chemistry breakthrough [138]. These solvents form through hydrogen bonding between natural components (choline chloride, sugars, organic acids) with dramatically lower toxicity compared to conventional organic solvents. For polyphenol extraction from spent coffee grounds using betaine:triethylene glycol (Bet:TEG) and choline chloride:1,2-propanediol (Chol:Prop), NADES proved as effective as conventional solvents while operating at milder temperatures without flammable solvents [141].

Most significantly, laboratory studies demonstrate that NADES-extracted bioactive compounds have 10-fold higher antimicrobial activity compared to ethanolic and aqueous extracts, suggesting structural preservation or enhancement during extraction [142].

Industrial Considerations: High viscosity and complex downstream separation are key challenges. Recycling and solvent removal can be energy-intensive at large scale, and regulatory approval for food-grade applications is still evolving. Process optimization, including water content adjustment, solvent formulation, and coupled purification strategies, is necessary for industrial adoption [143].

### 5.5. Pressurized Liquid Extraction (PLE)

Pressurized liquid extraction applies elevated temperature and pressure to enhance solvent penetration and compound recovery [5]. At the laboratory scale, for grape pomace valorization, PLE achieved higher yields (up to 79 g GAE/kg DW total phenols) compared to solvent extraction approaches (46.9 g GAE/kg DW), though with reduced compound diversity [144].

Industrial Considerations: High-pressure equipment entails capital investment and operational complexity. Excessive temperature or pressure may reduce compound diversity and degrade thermolabile bioactives. Pilot-scale continuous systems with precise pressure–temperature control are recommended to preserve bioactivity and ensure reproducibility at scale [145].

### 5.6. Supercritical Fluid Extraction (SFE)

Supercritical CO_2_ extraction eliminates the need for toxic organic solvents while protecting heat-sensitive compounds [142]. Laboratory studies for SCG demonstrate that supercritical and liquid CO_2_ extraction at 1 h achieved yields comparable to control methods requiring 5 h, with total polyphenolic contents of 970 mg GAE/100 g oil and enhanced antioxidant activities [129]. In laboratory studies, the technique also demonstrates particular advantages in the recovery of lipophilic compounds—particularly carotenoids from tomato by-products—where conventional organic solvent extraction proves problematic. Yields comparable to traditional approaches were achieved without requiring toxic organic solvents [142].

Industrial Considerations: Scaling SFE requires high-pressure vessels and precise process control, increasing capital and operational costs. The extraction of highly polar compounds may be limited without co-solvents, and batch heterogeneity can impact yield consistency [142]. Continuous SFE systems or hybrid SFE-DES approaches are often employed to balance yield, functionality, and industrial feasibility.

### 5.7. Integrative Perspective Linking Extraction Technologies and By-Product Valorization

The valorization pathways discussed in the preceding section—focused on FVR, BSG, and SCG—are intrinsically dependent on the selection and optimization of appropriate green extraction and bioprocessing technologies. The functional performance, safety, and industrial applicability of valorized ingredients such as pectins, proteins, polyphenols, dietary fibers, and lipids are strongly influenced by extraction conditions and solvent systems.

For FVR, mild physical-assisted techniques such as ultrasound-assisted and MAE are particularly effective in preserving the molecular integrity of pectins and polyphenols while reducing energy input and acid consumption [134].

In the case of BSG, EAE and fermentation-coupled processes enable the selective recovery of proteins and arabinoxylans with enhanced techno-functional and bioactive properties [126].

SCG, by contrast, benefit from advanced solvent-based and supercritical approaches that facilitate the efficient recovery of lipophilic compounds and high-potency antioxidants while minimizing thermal degradation [121].

Across all by-product streams, deep eutectic and natural DES emerge as unifying platforms capable of tunable, low-toxicity extraction, offering opportunities to harmonize sustainability goals with functional performance [137]. However, their successful implementation requires further advances in downstream processing, solvent recovery, and regulatory acceptance. Importantly, the comparative analysis highlights that no single technology is universally optimal; rather, matrix-specific and compound-targeted strategies—often combining physical, enzymatic, and solvent-based approaches—represent the most effective route to industrial-scale valorization.

Future research should therefore emphasize integrated process design, incorporating green extraction technologies within broader biorefinery frameworks aligned with formulation and application requirements. Such convergence is essential to translate the valorization concepts outlined previously into scalable, economically viable, and environmentally sound solutions that support circular food systems and climate-neutral processing.

## 6. Food Safety, Quality, and Regulatory Considerations

**Figure 3:**
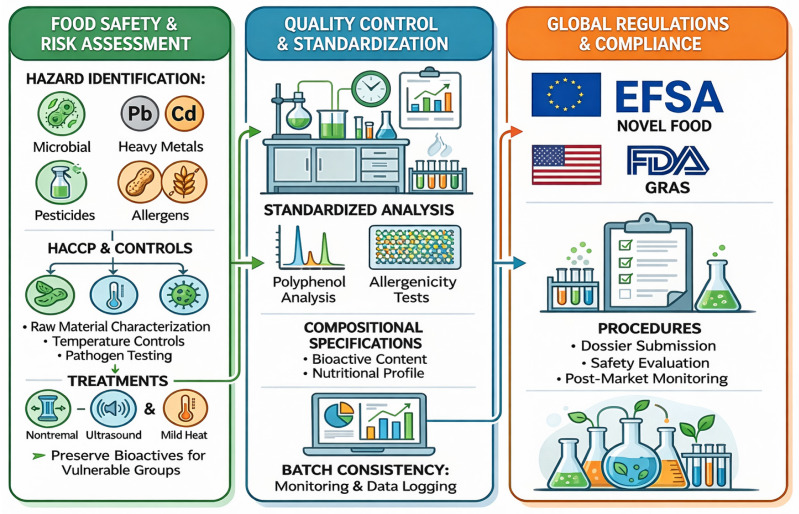
Food safety and compliance infographic.

Green extraction approaches offer environmentally friendly alternatives to conventional methods, yet their implementation necessitates adherence to stringent food safety standards, quality control protocols, and evolving regulatory frameworks (Figure 3).

Key quality and safety considerations include: residual solvent limits (particularly hexane and deep eutectic solvents), heavy metals and mycotoxins in agro-industrial streams, the standardization of polyphenol fingerprints for batch consistency, and protein allergenicity and digestibility assessments. Also, analytical standardization is critical to achieving regulatory approval [143].

### 6.1. Food Safety Considerations in Waste Valorization

#### 6.1.1. Risk Assessment and Hazard Identification

The development of functional food products from waste materials requires systematic risk assessment methodologies to ensure consumer safety [144,145]. Hazard identification must encompass biological, chemical, and physical hazards that may be present in food by-products feedstocks. Critical risks include microbial contamination from pathogenic microorganisms, chemical residues such as pesticides and heavy metals, and the presence of allergens [146].

The proper characterization of food residues as a raw material is essential, requiring assessment of its composition, bioactive compound content, and potential contaminants [147]. Heavy metal accumulation, pesticide residues, and mycotoxin contamination are significant safety concerns that vary depending on the agricultural practices used and environmental conditions where the waste originates [148]. Environmental monitoring and quality control of the raw material are fundamental requirements before extraction and processing [149].

#### 6.1.2. Microbiological Safety and Quality Control

Microbiological safety represents one of the most critical considerations in functional food production from waste materials [146]. Robust quality control systems must implement HACCP (Hazard Analysis and Critical Control Points) principles throughout production, as demonstrated in studies of fermented milk products and other functional foods [150]. All samples must be tested for pathogenic microflora including Salmonella, Listeria monocytogenes, and other foodborne pathogens [151].

Temperature control during extraction, processing, and storage is crucial for maintaining product safety and preventing microbial proliferation [150]. Processors can leverage both nonthermal and thermal reduction methods to balance safety with preservation of bioactive components [149]. Particularly for products intended for vulnerable populations, including infants and immunocompromised individuals, stringent hygiene protocols and microbiological testing at multiple production stages are mandatory.

#### 6.1.3. Chemical Contamination and Bioaccumulation

Chemical safety concerns in waste-derived functional ingredients include pesticide residues, heavy metals, and other contaminants that may bioaccumulate during the extraction and concentration process [151]. When recovering bioactive compounds from agricultural waste, testing for residues of pesticides, antibiotics, and other agricultural chemicals is essential [152]. The concentration effects of extraction processes may elevate residue levels, making raw material quality assessment particularly critical.

### 6.2. Quality and Regulatory Framework Considerations

#### 6.2.1. Global Regulatory Landscape

The regulatory status of functional foods and food supplements derived from waste valorization remains fragmented globally [153]. Different countries maintain divergent regulatory frameworks, with variations in standardization, ingredient approvals, and evidence requirements for functional claims [154]. In the European Union, the European Food Safety Authority (EFSA) evaluates the safety of novel foods and ingredients, requiring sponsors to submit information on multiple product batches [155]. The United States FDA similarly requires demonstration of GRAS (Generally Recognized as Safe) status for novel ingredients [156,157].

Ukraine and other countries are undertaking regulatory harmonization with international standards, though gaps remain in addressing emerging ingredients such as postbiotics with immunological activity [158]. The absence of harmonized definitions for functional foods across regions creates challenges for international trade and product standardization [158].

#### 6.2.2. Novel Food Authorization and Documentation

When turning food by-products into functional ingredients through extraction and processing, products may qualify as novel foods requiring authorization before market introduction [152]. Novel food determinations typically require comprehensive safety assessments, toxicological studies, and documentation of compositional analysis [159]. The regulatory pathway depends on the specific country and whether the ingredient demonstrates substantial equivalence to existing food components.

Regulatory advances increasingly acknowledge specific ingredients derived from agri-food residues. The EFSA has approved olive phenolics, citrus flavanones, and coffee cascara for specific health claims, illustrating the translational readiness of scientifically validated waste-derived ingredients [159]. However, regulatory approval typically requires submission of comprehensive scientific dossiers, including extraction method validation, compositional analysis, stability data, and safety assessment documentation.

#### 6.2.3. Quality Standards and Specifications

Establishing quality standards for functional ingredients derived from food by-products requires the development of comprehensive specifications addressing purity, potency, identity, and safety parameters [160]. The European Pharmacopoeia (Ph. Eur.) increasingly serves as a reference framework for supplement ingredient quality, encouraging food business operators to utilize pharmacopeial standards when assessing ingredient specifications [160].

Specifications must account for the batch-to-batch variability inherent in waste-derived materials. Unlike synthetic ingredients with consistent composition, bioactive compounds from plant-based food by-products may vary depending on seasonal factors, agricultural practices, processing conditions, and storage parameters [159]. The development of standardized extraction and processing protocols is essential to ensure consistency and facilitate regulatory approval.

## 7. Challenges, Limitations, and Future Directions

Hybrid extraction approaches combining multiple green technologies sequentially demonstrate enhanced efficiency compared to single-method approaches. However, standardized protocols enabling technology comparison and transfer across research groups remain underdeveloped.

### 7.1. Challenges, Limitations, and Knowledge Gaps

#### 7.1.1. Scalability and Standardization Issues

Despite promising research outcomes, scaling green extraction technologies from laboratory to industrial production faces significant barriers [5]. Equipment costs, solvent recovery complexities, and process standardization requirements remain primary constraints [161]. Deep eutectic solvent extraction, while demonstrating superior bioactivity preservation, requires further optimization for cost-effective large-scale implementation.

#### 7.1.2. Consumer Perception and Market Acceptance

The successful valorization of food by-products critically depends on consumer acceptance and market development [39]. While the technical feasibility of BSG incorporation into bread products is established, consumer surveys reveal concerns about darker appearance, lingering fiber particles, and aftertaste [162]. Educational initiatives emphasizing environmental sustainability and health benefits are essential for market expansion [163]. Consumer trust requires transparent communication regarding safety controls, quality assurance procedures, and regulatory compliance. Gen X consumers and older age groups express greater concerns regarding product quality and safety compared to younger demographic segments [163]. Effective market development requires stakeholder collaboration between practitioners, policymakers, and product developers to address consumer education gaps and build confidence in waste-to-value innovations.

#### 7.1.3. Regulatory and Safety Considerations

Current regulatory frameworks insufficiently accommodate food by-products, creating uncertainty for commercial development [164]. A decision tree framework suggested that successful valorization requires balancing sustainability, safety, and consumer relevance—with regulatory adaptation through private standards potentially accelerating development.

Pesticide residue monitoring becomes critical when valorizing fruit and vegetable by-products, particularly peel fractions accumulating agrochemicals [165].

#### 7.1.4. Environmental Impact Assessment

Artificial intelligence and machine learning approaches increasingly support the optimization of extraction parameters to maximize target compound yields while minimizing environmental impact [161,166]. A life-cycle assessment of by-product valorization processes remains essential for validating sustainability claims [5]. While green extraction techniques demonstrate reduced solvent consumption and energy requirements compared to conventional methods, a comprehensive environmental impact analysis encompassing full production chains is necessary for regulatory compliance and stakeholder confidence.

### 7.2. Future Perspectives

#### 7.2.1. Commercial Viability and Market Development

Product development from by-product-derived bioactive compounds requires parallel advancement in three domains: technological optimization, regulatory adaptation, and consumer education [167]. Successful market entry demands the identification of optimal BSG incorporation levels (5–15% for sensory acceptance), development of innovative food formulations, and transparent communication of sustainability benefits.

#### 7.2.2. Alignment with Sustainable Development Goals

Food by-products valorization directly supports UN Sustainable Development Goals 9 (Industrial Innovation and Infrastructure), 12 (Responsible Consumption and Production), and 13 (Climate Action), among others [167]. The integration of valorization strategies within food systems simultaneously promotes environmental protection, economic development, and social equity.

## 8. Conclusions

FVR, BSG, and SCG represent compositionally rich and underutilized resources. Advances in green extraction and integrated biorefinery design are enabling the transformation of these residues into functional ingredients for food applications.

The transformation of FVR, BSG, and SCG into functional food ingredients through green extraction approaches represents both scientific advancement and practical necessity. Recent developments demonstrate the technical feasibility, economic viability, and environmental benefits of valorization strategies. UAE, MAE, SFE, and DES/NADES emerge as the most promising green extraction technologies, with research confirming superior bioactive compound preservation compared to conventional methods.

BSG protein isolates, coffee polyphenols, and fruit polysaccharides exhibit nutritional and functional properties supporting their incorporation into diverse food matrices at optimization levels maintaining sensory acceptability. Fermentation-based bioprocessing enhances nutritional profiles while creating a platform for enzyme and metabolite production, enabling integrated biorefinery approaches.

However, successful commercialization requires coordinated advancement in three areas: (1) the standardization of green extraction protocols enabling industrial scale-up, (2) regulatory framework adaptation accommodating food by-products, and (3) consumer education emphasizing environmental and health benefits. Continued progress requires coordinated research across the fields of food chemistry, bioprocess engineering, safety science, and market policy to establish standardized, scalable, and economically viable valorization pathways.

## Figures and Tables

**Table 1:** Composition of key food processing co-products (representative ranges).

By-Product	Major Components	Typical Composition (Dry Basis)	References
Fruit and Vegetable Pomace	Pectin, soluble fiber, polyphenols	Ash/Minerals 3–8%	[25,26,27,28]
		Carbohydrates 40–60%	
		Lipids 2–10%	
		Proteins 8–15%	
		Total fiber 40–60%	
Fruit and Vegetable Peels	Polyphenols, pectin fiber	Ash/Minerals 3–13%	[29,30,31,32,33,34]
		Carbohydrates 10–50%	
		Lipids 1–5%	
		Proteins 2–10%	
		Total fiber 20–60%	
Fruit and Vegetable Seeds	Proteins, lipids, lycopene	Ash/Minerals 3–5%	[35,36,37]
		Carbohydrates 20–45%	
		Lipids 15–65%	
		Proteins 10–25%	
		Total fiber 10–55%	
Fruit and Vegetable Pulp	Carbohydrates, vitamins, fibers	Ash/Minerals 1–5%	[36,38]
		Carbohydrates 30–70%	
		Lipids 0.1–30%	
		Proteins 1–5%	
		Total fiber 2–15%	
Brewer’s Spent Grain	Hemicellulose, cellulose, proteins, phenolic acids	Protein 15–30%;	[39,40,41]
		Total fiber 50–70%;	
		Lipids 5–10%	
Spent Coffee Grounds	Cellulose, hemicellulose, lignin, lipids, melanoidins	Lipids 10–18%;	[42,43,44]
		Total fiber 60–70%;	
		Phenolics 1–3%	

**Table 2:** Food applications of valorized ingredients.

Food Application	Ingredient Extracted	Source	Key Functionality	References
Jams, yogurts, beverages	Pectin	FVR (pomace and peels)	Gelling, thickening, stabilizing	[54,55,56,57]
Bakery, plant-based meats	Protein concentrates	BSG	Emulsification, foaming, gelling	[58,59,60,61]
Emulsions, antioxidants	Polyphenol extracts	FVR (pomace and peels) and SCG	Oxidative stability, color retention	[62,63,64,65,66,67,68,69,70]
Bakery, meat analogs	Dietary fibers	SCG and FVR (peels and pulp)	Water retention, texture modification	[71,72,73,74,75,76]

**Table 3:** Comparison of the most frequently applied green extraction technologies.

Method	Advantages	Limitations	Typical Applications/Target Compounds	References
UAE	Low temperature; reduced extraction time	Scale-up complexity; equipment cost	Pectins, polyphenols, proteins from FVR and BSG; antioxidants	[131,132,133,134]
MAE	High efficiency; low solvent usage	Risk of thermal degradation	Polysaccharides, pectins, robust phenolics; some proteins	[134,135]
EAE	High selectivity; mild conditions	Enzyme cost; process optimization required	Protein hydrolysates, bioactive peptides, soluble fibers from BSG and FVR	[136]
DES/NADES	Tunable, low-toxicity solvents	High viscosity; challenging downstream separation	Polyphenols, pectins, flavonoids from SCG, FVR; functional ingredients	[137]
PLE	High extraction efficiency; reduced solvent use	High capital cost; reduced compound diversity	Polyphenols and antioxidants from grape pomace, FVR	[138,139]
SFE	Solvent-free for lipophilic compounds; protects heat-sensitive compounds	High equipment cost; complex operation	Lipids, carotenoids, caffeine, and other lipophilic bioactives from SCG and tomato by-products	[121]

## Data Availability

No new data were created or analyzed in this study. Data sharing is not applicable to this article.

## Abbreviations

The following abbreviations are used in this manuscript:

FVR	Fruit and vegetable residues
BSG	Brewer’s spent grain
SCG	Spent coffee grounds
UAE	Ultrasound-assisted extraction
MAE	Microwave-assisted extraction
EAE	Enzyme-assisted extraction
DES	Deep eutectic solvents
PLE	Pressurized Liquid Extraction
SFE	Supercritical Fluid Extraction
